# The platelet derived growth factor-B polymorphism is associated with risk of severe fever with thrombocytopenia syndrome in Chinese individuals

**DOI:** 10.18632/oncotarget.9043

**Published:** 2016-04-27

**Authors:** Xiao-Ai Zhang, Chen-Tao Guo, Qing-Bin Lu, Jian-Gong Hu, Ning Cui, Zhen-Dong Yang, Wei Peng, Rong Liu, Chun-Yan Hu, Shu-Li Qin, Xian-Jun Wang, Shu-Jun Ding, Dou-Dou Huang, Wei Liu, Wu-Chun Cao

**Affiliations:** ^1^ State Key Laboratory of Pathogen and Biosecurity, Beijing Institute of Microbiology and Epidemiology, Beijing, 100071, P. R. China; ^2^ Graduate School of Anhui Medical University, Hefei, 230032, P. R. China; ^3^ School of Public Health, Peking University, Beijing, 100191, P. R. China; ^4^ The 154 Hospital, People's Liberation Army, Xinyang, 464000, P. R. China; ^5^ The Shangcheng People's Hospital, Xinyang, 464000, P. R. China; ^6^ Shandong Provincial Center for Disease Control and Prevention, Jinan, 250001, P. R. China

**Keywords:** severe fever with thrombocytopenia syndrome, polymorphism, susceptibility, PDGF-B, cytokines

## Abstract

**Background:**

Severe fever with thrombocytopenia syndrome (SFTS) is an emerging infectious disease caused by a novel bunyavirus named SFTS virus (SFTSV). We hypothesize that host genetic variations may contribute to susceptibility to SFTS.

**Results:**

Compared with the rs1800818 AA genotype, AG + GG genotypes were significantly associated with increased susceptibility to SFTS (odds ratio, 1.66, 95% confidence interval = 1.28-2.16; *P* < 0.001). By using the ELISA assay, we observed that PDGF-BB concentration was significantly reduced in acute phase of patients than in the controls (*P* < 0.001) and recovered patients at 6 month (*P* = 0.007) and 12 month (*P* = 0.003). A persistently reduced PDGF-BB was also revealed from the SFTSV-infected C57BL/6J mice (*P* < 0.001). The rs1800818 G allele was associated with decreased serum PDGF-BB levels in SFTS patients at their early infection (*P* = 0.015). In accordance, the relative mRNA levels of the at-risk G allele of 1800818 were lower than those of the A allele in heterozygous cell from acute phase of SFTS patients. *PDGF-B* rs1800818 conferred no susceptibility to severe or fatal outcome in SFTS patients.

**Materials and Methods:**

An initially small-scale case-control association study guided the selection of platelet derived growth factor-B (*PDGF-B*) rs1800818 in 1020 SFTS patients and 1353 controls. Functional analyses were conducted to verify the biological significance of rs1800818 polymorphism.

**Conclusions:**

Our findings suggest that the *PDGF-B* rs1800818 polymorphism might play a role in mediating the susceptibility to SFTS.

## INTRODUCTION

Severe fever with thrombocytopenia syndrome (SFTS) is an emerging infectious disease that is caused by a novel bunyavirus named SFTS virus (SFTSV) in the *Bunyaviridae* family. Since its discovery in 2009 in the middle east of China, SFTSV infection has been documented in 19 provinces, leading to at least 2047 reported cases nationally in 2011 and 2012 [1]. Recently SFTSV infected cases were also reported from South Korea and Japan, indicating the imminent public health impact of this emerging infectious disease [2-4]. The SFTSV infected patients had wide clinical spectrum, with some experiencing self-limiting clinical course, while approximately 12% might develop fatal disease. The iceberg phenomenon of SFTS is obvious that a seroprevalence of 0.84-6.37% was found among the healthy population residing in endemic areas [5, 6]. Although with large amount of individuals who had been exposed to the virus, only a small proportion developed symptomatic disease, suggesting the role of host susceptibility in determining the clinical outcome. Research on person to person transmission events disclosed a higher tendency of having clinical disease and severe disease outcome in the inheritable related individuals, providing further evidence that human genetic background might play roles in the disease development and modulation of disease severity [7, 8].

The pathogenesis of SFTS has been partially explicated, indicating remarkable virus replication and a dyregulated immune response after infection contributing to disease progression [9, 10]. Among all the immune dyregulation, the cytokine mediated inflammatory response, characterized by cytokine/chemokine production imbalance, was found to be responsible for SFTSV infection and disease progression [9-11]. It makes sense to hypothesize that some genetic determinants might reside in those immune system genes polymorphisms that regulate host immune responses, in particular cytokine gene polymorphisms. Up to the current stage, although a handful of cytokine/chemokine has been evaluated in SFTS patients, most of the results have been inconsistent [9-11]. The only one exception was platelet derived growth factor-BB (PDGF-BB), which had been independently and consistently observed to be decreased in the SFTS patients than controls, regardless of the sample size or sampling time in different studies [10, 12]. PDGF is a family of cationic homo- and heterodimers of disulfide-bonded A- and B- polypeptide chains, acting as the major growth factor in health and disease. The BB isoform of PDGF (PDGF-BB) is a key regulatory molecule in various physiological processes such as bone homeostasis, repair and regeneration [13]. The effects of PDGF family on endothelial cells have been demonstrated in various studies [14, 15]. Since SFTSV can infect vascular endothelial [16], it's highly possible that PDGF might play a role in the pathogenesis of the SFTSV infections.

For the *PDGF-B* gene, the population based association studies have been focused on two single nucleotide polymorphisms (SNPs), rs1800818 located in the 5b 2-UTR and rs1800817 in first intron [17-21]. The rs1800818, a isoleucine (Ile) to valine (Val) substitution, was found to affect receptor affinity and cell activation [17]. The rs1800818 and rs1800817 polymorphisms have been demonstrated to alter the individual risk to cardiac allograft vasculopathy [18]. It has been shown that the rs1800818 polymorphism was the leading SNP that predicted 3-year overall survival in patientas with resected colorectal liver metastases who receive bevacizumab-based chemotherapy [19]. An association between the *PDGF-B* rs1800817 polymorphism and severe recurrent HCV infection after liver transplantation was also observed [21]. The role of the *PDGF-B* polymorphisms in SFTS, however, has never been investigated. In the present study, we examined whether these two important *PDGF-B* polymorphisms has any bearing on the risk or severity of SFTS in Chinese populations.

## RESULTS

### Clinical and laboratory characteristics of study subjects

A total of 1020 virologically confirmed SFTS patients and 1353 controls were recruited for the study. By checking the medical records and by interviewing the participants, we determined that all cases and controls were genetically unrelated Han Chinese. The selected characteristics of subjects are shown in Table 1. Compared with the controls, the SFTS patients had significantly older age (*P* < 0.001), higher frequency of female (*P* < 0.001) and more presence of underlying conditions (*P* = 0.013).

**Table 1:** Selected characteristic of patients with severe fever with thrombocytopenia syndrome and controls

Variables	Patients					Controls (n= 1353)	*P* value^b^		
	Total (n = 1020)	Mild patients (n= 730)	Severe patients (n= 290)	Nonfatal patients (n= 912)	Fatal patients (n= 108)		Patients vs. Control	Mild vs. Severe patients	Nonfatal vs. Fatal patients
Age, year									
Mean (SD)	60.7 (12.0)	59.8 (12.1)	62.7 (11.7)	59.8 (12.0)	67.5 (10.4)	47.8(19.2)	< 0.001	< 0.001	< 0.001
≤ 60, n. (%)	473 (46.4)	361 (49.5)	112 (38.6)	453 (49.7)	20 (18.5)	972 (71.8)	< 0.001	0.002	< 0.001
Male, n (%)	428 (42.0)	280 (38.4)	148 (51.0)	373 (40.9)	55 (50.9)	694 (51.3)	< 0.001	< 0.001	0.046
Underlying medical conditions^a^, n (%)	278 (27.3)	171 (23.4)	107 (36.9)	234 (25.7)	44 (40.6)	309 (22.8)	0.013	< 0.001	0.001

Abbreviations: SD, standard deviation.

a The underlying medical conditions were defined as patients presenting with one of the following: hypertension, diabetes, cancer, active hepatitis, cerebral infarction, et al.

b *χ*^2^ test for categorical variables and the Mann Whitney U test for continuous variable

### *PDGF-B* rs1800818 *G* allele conferred increased susceptibility to SFTS disease

**Figure 1:**
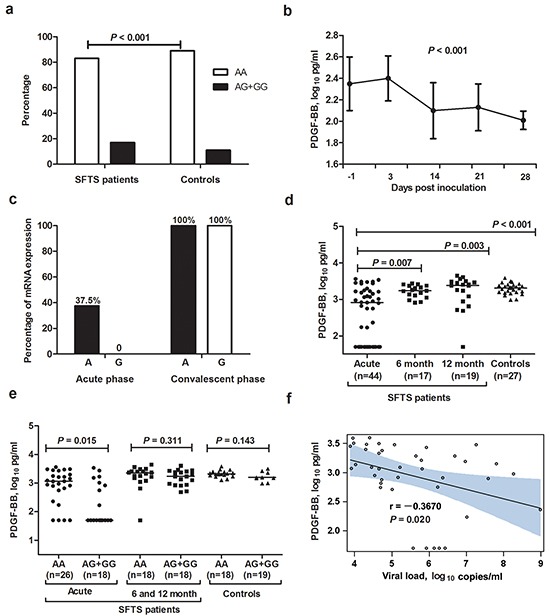
*PDGF-B* rs1800818 polymorphism and PDGF-BB expression in severe fever with thrombocytopenia syndrome patients and controls **a.** Proportion of all SFTS patients and controls carrying rs1800818 AA or AG+GG genotype. **b.** Dynamic profile of PDGF-BB in sera of SFTSV-infected C57BL/6J mice. The generalized linear model was used to calculate the linear trend of the mouse PDGF-BB over time. **c.** Allele-specific expression of *PDGF-B* messenger RNA in the paired peripheral blood mononuclear cells from SFTS patients. **d.** Serum PDGF-BB levels in SFTS patients at acute phase and recovered SFTS patients at 6 and 12 months after disease, in comparison with controls. **e.** Serum PDGF-BB levels in SFTS patients, recovered SFTS patients (at 6 month and 12 month) and controls with rs1800818 AA and AG+GG genotypes. **f.** Correlation between PDGF-BB levels and SFTSV load in serum of SFTS patients.

The initial small-scale association study was performed in the first cohort of 250 SFTS patients and 250 controls. The genotyping results were presented in Supplementary Table S2. The observed genotype frequencies of the rs1800818 and rs1800817 polymorphisms were in Hardy-Weinberg equilibrium in both patients and controls groups (all *P* > 0.05, data not shown). By using multivariate logistic regression model to adjust for the effect from age, sex, and underlying disease, significant associations with SFTS were observed for the *PDGF-B* rs1800818. The association between rs1800818 and SFTS remained after multiple corrections (Supplementary Table S2). There was no association between rs1800817 polymorphism and SFTS (Supplementary Table S2). Therefore we focused on rs1800818 for further study. With DNA samples from more patients available over time, we genotyped rs1800818 from additional 770 SFTS patients and 1103 controls by PCR-direct sequencing, which results were combined with those obtained from initial small-scale association study for analysis. In all of the 1020 SFTS patients and 1353 controls, rs1800818 G allele was significantly overrepresented in SFTS patients than controls (9% vs. 5.6%, *P* < 0.001). After adjustment for age, sex, and underlying medical conditions, the genotypes containing G allele (AG + GG genotypes) were significantly associated with increased susceptibility to SFTS (OR = 1.66, 95% CI = 1.28-2.16; *P* < 0.001; Table 2, Figure 1a).

**Table 2:** Association of *PDGF-B* rs1800818 polymorphism with severe fever with thrombocytopenia syndrome

Genotypes	Patients (n = 1020)	Controls (n = 1353)	Model	OR (95% CI)^a^	*P* value^a^
AA	799 (83.1)	1115 (82.4)		Reference	
AG	151 (15.7)	135 (10.0)	Codominant	1.57 (1.20-2.05)	< 0.001
GG	11 (1.1)	3 (0.2)		6.18 (1.59-23.98)	
AG + GG	162 (16.9)	138 (10.2)	Dominant	1.66 (1.28-2.16)	< 0.001
			Recessive	5.80 (1.50-22.48)	0.005
			Overdominant	1.55 (1.18-2.03)	0.001
			Log-additive	1.68 (1.31-2.15)	< 0.001

Abbreviations: OR, odds ratio; CI, confidence interval;

a The ORs and *P* values were adjusted for age, sex, and underlying medical conditions.

### PDGF-BB was persistently reduced in SFTSV-infected C57BL/6J mice

Altogether 50 mice were successfully infected with SFTSV; the viral loads of the serum samples were shown to be maintained at detectable level throughout the whole observation, indicating an effective infection with SFTSV. Post infection, the reduction of PDGF-BB levels was observed at first observation on 3 DPI, which kept decreasing in the serially collected serum samples on 14 and 21 DPI, till the last observation of 28 DPI (*P* < 0.001, Figure 1b).

### Effects of the rs1800818 polymorphism on PDGF-BB expression in SFTS patients and controls

For eight rs1800818 heterozygous (GA genotype) patients who had paired PBMC estimated, at the acute phase, three had mono-allelic (A allele) *PDGF-BB* expression, however, G allele related *PDGF-BB* expression was undetectable for all patient. Furthermore, PDGF-B messenger RNA was increased at the convalescent phase and we observed that bi-allelic *PDGF-B* expression related to rs1800818 polymorphism in the PBMCs from the convalescent phase of all eight patients (Figure 1c).

Altogether 44 SFTS patients and 27 controls were evaluated for the serum PDGF-BB level. The serum PDGF-BB levels from SFTS patients at acute phase were significantly lower than that obtained from controls (*P* < 0.001, Figure 1d). In addition, the PDGF-BB levels from the recovered SFTS patients were significantly increased to comparable level with controls at 6 moths (*P* = 0.219) and 12 month (*P* = 0.577) after disease. Among the 44 SFTS patients, those carrying the rs1800818 AG+GG genotype (n=26) had significantly lower PDGF-BB level than the AA genotype carriers at acute phase (n=18) (*P* = 0.015, Figure 1f). However, no such difference was observed among either controls or recovered SFTS patients at 6 and 12 months after disease (Figure 1e). At acute phase, PDGF-BB levels were also negatively correlated with SFTSV loads (*P* = 0.020, Figure 1f).

### *PDGF-B* rs1800818 conferred no susceptibility to severe outcome in SFTS patients

**Figure 2:**
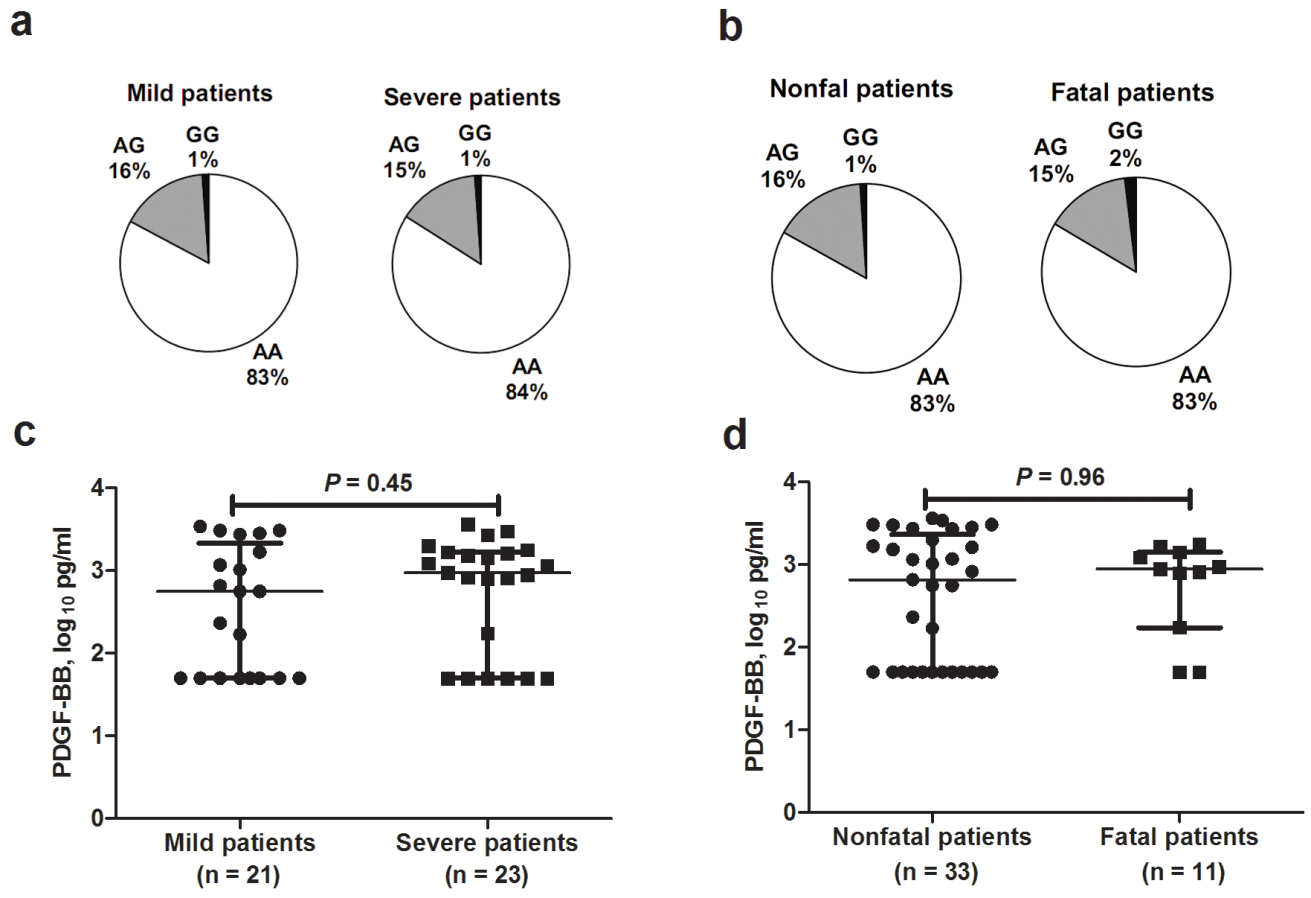
*PDGF-B* rs1800818 and PDGF-BB expression among severe fever with thrombocytopenia syndrome patients with different outcomes **a.** Genotype frequencies obtained from severe fever with thrombocytopenia syndrome (SFTS) patients with severe and mild disease. **b.** Genotype frequencies obtained from SFTS patients with fatal and nonfatal outcome. **c.** Serum PDGF-BB levels between mild SFTS patients and severe SFTS patients. **d.** Serum PDGF-BB levels between SFTS patients with fatal and nonfatal outcome.

Among the 1020 SFTS patients, 290 developed severe disease outcome, comprising 108 fatal cases. Compared with mild patients, significantly older age, more male gender and presence of underlying medical conditions were found in severe patients (Table 1). Similar results were found when comparison was made between fatal and nonfatal patients (Table 1). Comparison between severe patients vs. mild patients (OR = 0.87, 95% CI = 0.59-1.29; *P* = 0.48; Figure 2a and Supplementary Table S3) and fatal patients vs. nonfatal patients (OR = 0.91, 95% CI = 0.51-1.62; *P* = 0.75; Figure 2b and Supplementary Table S4) displayed no significant difference of the genotype frequencies for *PDGF-B* rs1800818 polymorphism after adjusting the effect from age, gender and underlying medical conditions by applying multivariate regression analysis. The PDGF-BB levels that were measured at acute infection in severe cases were comparable with mild cases (*P* = 0.45; Figure 2c). The PDGF-BB levels that were measured at acute infection in fatal cases were comparable with non-fatal cases as well (*P* = 0.96, Figure 2d).

### *PDGF-B* rs1800818 exerted minor effect on clinical recovery in SFTS patients

We also analyzed the effect of rs1800818 polymorphism on clinical recovery of SFTS patients, with important laboratory parameters that were serially evaluated as dependent variables, i.e, PLT (platelets), WBC (white blood cell), ALT (alanine transaminase), AST (aspartate aminotransferase), LDH (lactate dehydrogenase) and viral load (Supplementary figure S1). As we have displayed for all patients, serial LDH and viral loads demonstrated significantly differential pattern for the genotypes containing G allele (AG + GG genotypes) compared with the AA genotype (Supplementary Figure S1a and S1d). Both evaluations were kept at comparable level between two groups of patients at early phase of infection, however on 7-9 days after disease when patients entered into convalescent phase, patients who carried AG + GG genotypes demonstrated more rapid viral clearance (*P* = 0.009) and recovery of LDH to normal level (*P* = 0.03). No significant difference was observed for other laboratory parameters regarding the rs1800818 genotypes (Supplementary Figure S1b, S1c, S1e and S1f).

## DISCUSSION

It is well-known that host genetic immunity might determine the outcome of infectious diseases. In this study, we found that PDGF-BB secretion was significantly reduced at acute phase of SFTS patients and *PDGF-B* rs1800818 polymorphism was significantly associated with host susceptibility to SFTS. Genotypes containing G allele confer increased risk of SFTS disease than the AA genotype. The G allele-specific *PDGF-B* RNA expression and PDGF-BB serum levels were significantly lower than those derived from A allele or AA genotype. In mice model, the SFTSV infected mice demonstrated a consistently decreased expression of PDGF-BB. Taken this together, we provided evidence that PDGF-BB might be associated with SFTS disease at both the genetic and serum levels. It's suggested that the ability of individuals to respond properly to SFTSV infection may be impaired by SNPs within *PDGF-B* gene, resulting in decreased transcription and secretion of PDGF-BB, eventually leading to an increased susceptibility to SFTS development.

**Figure 3:**
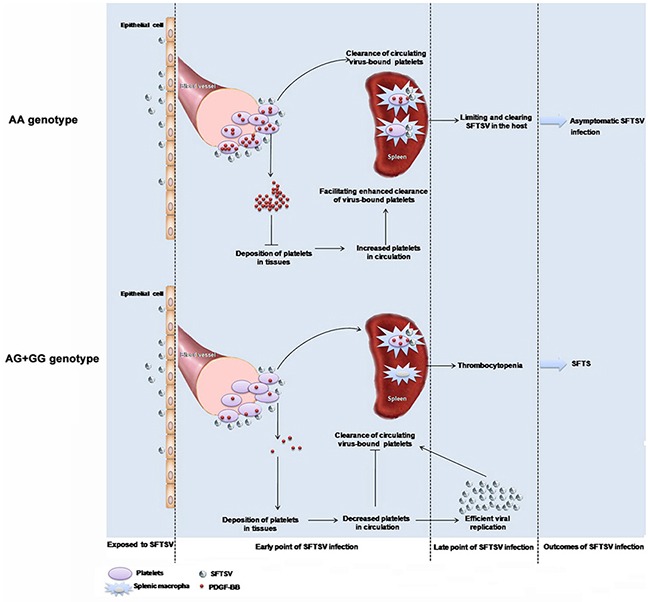
Proposed mechanism of *PDGF-B* rs1800818 polymorphism impacting on severe fever with thrombocytopenia syndrome virus infection

The observed genetic association is plausible from a biological perspective. Platelet is the fundamental component of primary hemostasis, which is also known to release many growth factors when aggregated and activated, including PDGF, transforming growth factor-beta, vascular endothelial growth factor and epidermal growth factor. The PDGF released from platelets, on the other hand, serves an autocrine feedback role in control of platelet aggregation. Thrombocytopenia is the major clinical hallmark symptom of SFTSV infection, the underlying pathogenic mechanism is suggested to be clearance of circulating virus-bound platelets by splenic macrophages, as displayed in C57/BL6 mouse model [9]. In case of SFTSV infection, SFTSV adherence on platelets resulted in enhanced release of PDGF from platelets, exerting feedback control effects on platelet aggregation. This further lead to decreased deposition of platelets in tissues and increased platelets in circulation, facilitating the enhanced clearance of virus-bound platelets promoted by splenic macrophages. This process represents the host response of limiting and clearing SFTSV in asymptomatic individuals. In contrast, incapability of PDGF-BB express and release in individuals who carried the rs1800818 *G* allele might be associated with suppressed PDGF-BB regulation on SFTSV clearance, which ultimately lead to active viral replication and thrombocytopenia, manifesting as the SFTS (Figure 3). This could be evidenced by depressed PDGF-BB expression from both human specimens and infected mice in the current study. The significant inverse correlation between PDGF-BB and SFTSV viral loads provided further evidence. As the disease progress into convalescence phase, when the virus began to be cleared by activated immunity, the genotype specific effect from SFTSV was suggested to decline, finally eliciting a rapid recovery of PDGF-BB level, as well as a more rapid clinical recovery of these patients. This genotype specific effect on PDGF-BB expression was not observed in healthy control or the recovered patients, which supported our hypothesis that the transcriptional regulation of PDGF-BB might take place at SFTSV viral exposure and early than the development of the thrombocytopenia. Although with this biologically based priori hypothesis, it remained obscure how the regulation of PDGF-BB induction on virus binding occurred at the transcriptional level. The mechanism underlying the potential autocrine feedback effect of the PDGF-BB in control of platelet aggregation remained to be further investigated.

On the other hand, we failed to find association between rs1800818 polymorphism and severe outcome in SFTS patients, from either genetic level or serum level, indicating minor role of PDGF-BB in determining severe outcome in SFTS patients. As have been indicated in mouse model, virus replication and over exuberant immune responses contribute to the progressive organ damage resembling human SFTS [9]. The effect of PDGF-BB on severe outcome in SFTS patients, if there is any, might be masked by other uninvestigated host immune related factors. On the other hand, the small sample size of fatal case has hindered our efforts in identifying potential associations between genetic factor and disease outcome, which need further investigation on another case cohort.

The current study has the advantage of bearing desirable features that are considered as components of an ideal genetic association study. These characteristics include rigorous case selection, associations that make biological sense and allele that affect the gene product in a physiologically meaningful way. An extraordinary advantage of this study is the minimal interference from the recall bias that is inherent to the traditional case control study. As is known, SFTS is an emerging infectious disease that had become endemic since 2010, which had attracted intense attention in local residence due to the high case fatality rate. Therefore the previous medical history that resembled the SFTS is unlikely to be missed by the recalling information of questionnaire interview. The positive IgG detection in endemic region is highly indicative that patients never had the clinical disease after exposure to SFTSV.

In conclusion, our data provide strong evidence that the *PDGF-B* rs1800818 secretion regulated by genetic polymorphism may affect the occurrence of SFTS in Chinese population. The screen of this risk allele in SFTS endemic region might help to identify individuals with high-risk of becoming ill after exposure to SFTSV, for strengthened prevention measures to be taken. From clinical perspective, the specifically depressed PDGF-BB in SFTS has been suggested in our study, supporting its potential application as clinical diagnosis marker. Until recently, no specific therapy is available to treat this infection with high morbidity. The current finding, if confirmed in other cohorts, might offer perspective of applying PDGF-BB as a valuable therapeutic target. Moreover, these findings could shed light on the pathogenesis of SFTS, if PDGF-BB and its signaling pathway involvement could be further explored. Although with this advantage, our initial findings should be independently verified in populations of different ancestry, in case of adequate patients sample is accessible.

## MATERIALS AND METHODS

### Study subjects and information collection

The study was performed in the SFTS designated hospital (The PLA 154 Hospital and The Shangcheng People's Hospital) in Xinyang administrative district of Henan Province between 2014 and 2015. On admission, all clinical diagnosed SFTS patients had sera samples collected and subjected to SFTSV RNA test by a molecular method as described previously (detailed in supplemental material and methods) [22]. Totally 1020 patients with a positive result were invited to participate in the study. Severe cases were defined by the presence of hemorrhagic manifestations (epistaxis, hematemesis, and melena), or presence of one or more organ failure or encephalitis development [23].

Altogether 1353 controls were randomly selected from healthy subjects who underwent routine physical examination in the same region during the same period that the cases were recruited. At recruitment, informed consent was obtained from all participants, and personal information on demographic factors and medical history were collected via structured questionnaire. The study was performed with the approval of the Ethical Committee of Beijing Institute of Microbiology and Epidemiology and conducted according to the principles expressed in the Declaration of Helsinki.

### Genotyping of rs1800818 and rs1800817 polymorphisms

Two SNPs of *PDGF-B* (rs1800818 and rs1800817) that either have reputed functional significance or been reported to affect disease susceptibility were evaluated in the present study [18, 21]. The initial small-scale case-control study involving the determination of rs1800818 and rs1800817 polymorphisms in 250 SFTS patients and 250 controls were analyzed by the MassArray System (Sequenom) with primers (see Supplementary Materials, Supplementary Table S1) as described previously [24]. Additional 1873 DNA samples were genotyped for only rs1800818 by polymerase chain reaction (PCR) direct sequencing (detailed in supplemental material and methods). Genotyping was done in a blind manner that the performers did not know the subjects' case and control status. For quality control, a 15% masked, random sample of cases and controls was tested twice by different people and all results were 100% concordance. In addition, genotypes identified by the MassArray System were confirmed by DNA sequencing.

### PDGF-BB expression in SFTSV-infected C57BL/6J mice

To evaluate the expression of PDGF-BB after SFTSV infection, C57BL/6J mice of 3- to 4-week-old were challenged by intraperitoneal injection with 3×10^7^ focus forming unit (FFU) of SFTSV. Every 6-7 mice were bleeded on day 3, 14, 21 and 28 post infection (DPI) (detailed in supplemental material and methods). The PDGF-BB level at each time point was detected by ELISA Kit (R&D Systems Inc., USA). All measurements were performed in duplicate. All experimental procedures with animals were in strict accordance with the recommendations in the Guide for the Care and Use of Laboratory Animals and approved by the Committee on the Ethics of Animal Experiments of the Academy of Military Medical Sciences.

### *PDGF-B* messenger RNA expression in SFTS patients

The rs1800818 polymorphism lies in the 5b 2-UTR of the *PDGF-B* gene. This allowed us to determine allele-specific gene expression of *PDGF-B* messenger RNA in rs1800818 heterozygous (GA genotype) individuals. Paired acute and convalescent peripheral blood mononuclear cells (PBMCs) were collected from 8 SFTS patients and measured for the *PDGF-B* messenger RNA using real-time quantitative PCR with G allele-specific probe and *A* allele-specific probe, respectively (detailed in Supplementary Materials).

### PDGF-BB expression in serum samples of SFTS patients and controls

To compare the expression patterns of PDGF-BB, acute SFTS patients and age matched controls were selected to measure the PDGF-BB levels by using PDGF-BB ELISA assay (GenWay Biotech, USA). The SFTS patients who were successfully followed up and sampled during the convalescence were also tested for the PDGF-BB evaluation. All measurements were performed in duplicate.

### Statistical analysis

Genotype and allele frequencies for polymorphisms were determined by gene counting. The fitness to the Hardy-Weinberg equilibrium was tested using the *χ*^2^ test. Associations between polymorphisms and risk of SFTS were estimated by use of logistic regression analyses. Odds ratios (ORs) and 95% confidence intervals (CIs) were used to measure the strength of association. In view of the multiple comparisons, the correction factor n (m–1) (n loci with m alleles each) was applied to correct the significance level. The PDGF-BB concentrations were log transformed, and tested for differences between different groups by two-sample Wilcoxon rank-sum test. These analyses were performed using SPSS software (version 17.0, SPSS Inc., Chicago, IL).

## SUPPLEMENTARY FIGURE AND TABLES


