# Supplementary material for: The platelet derived growth factor-B polymorphism is associated with risk of severe fever with thrombocytopenia syndrome in Chinese individuals
Source: Oncotarget. 2016 Apr 27;7(22):33340–9. doi: 10.18632/oncotarget.9043 (PMC5078099; doi:10.18632/oncotarget.9043)
Supplement: Supplementary file 1 [file oncotarget-07-33340-s001.pdf]

SUPPLEMENTARY MATERIALS

Severe fever with thrombocytopenia syndrome virus (SFTSV) RNA detection and concentration determination

Viral RNA was isolated from serum samples using QIAamp Viral RNA Mini Kit (Qiagen, Germantown, MD, USA), according to the manufacturer's instructions. One step Primer Script RT-PCR Kit (TaKaRa) was used according to the manufacturer's instructions for SFTSV detection according to the method described previously [1]. The reaction was performed on an ABI 7500 Real-Time PCR System (Applied Biosystems, USA). The primers and TaqMan probes used for the SFTSV detection were as follows: 5′-TTCACAGCAGCATGGAGAGG-3′ (forward primer), 5′-GATGCCTTCACCAAGACTATCAATG-3′ (reverse primer), 5′-AACTTCTGTCTTGCTGGCTCCGC-3′ (probe).

Quantitation of virus in sera from 44 SFTS patients was performed using quantitative RT-PCR targeting the same gene segments. In brief, plasmids containing a known copy number of amplification targets were included in the real-time PCR assay to generate a standard curve for quantification of test samples. Positive and negative controls were included. Standard curves included 5 dilutions and 3 replicate wells for each dilution. All samples were quantified in at least duplicate wells. Levels of SFTSV RNA concentrations were expressed as copies/ml.

Genotyping of rs1800818 polymorphism

A total of 1020 virologically confirmed SFTS patients and 1353 controls were genotyped for the rs1800818 polymorphism. In the initial small-scale case-controls study involved 250 SFTS patients and 250 controls, the rs1800818 polymorphism was analyzed by the MassArray System (Sequenom) with primers (Table S1) as described previously [2].

Additional 1873 DNA samples (770 SFTS patients and 1103 controls) were genotyped for rs1800818 by polymerase chain reaction (PCR) direct sequencing. The primer for the target region was designed using the Web-based software Primer 3.0 (available at: http://frodo.wi.mit.edu/primer3/). The primers 5′-cgctgcaaagagaaaaccg-3′ and 5′-cagggagaggtgcaaact-3′ were used for amplifying the target region. The PCR reactions were performed in GeneAmp PCR System 9700. Briefly, each 25 µL of PCR mixture contained 20 ng DNA, 0.2 µM of each primer, 0.2 mM deoxynucleoside triphosphates, 0.625 unit Tks Gflex DNA Polymerase (Takara BioTech, Dalian, China), and 1 × reaction buffer. The PCR conditions were performed as described previously except for an annealing temperature of 60°C [3].

SFTSV challenge in mice

Seventy female C57BL/6J mice of 3- to 4-week-old were purchased from the Laboratory Animal Center of PLA Military Academy of Medical Sciences. Before challenge, 14 mice were sacrificed and their fresh blood, livers, spleens and kidneys were collected as pre-challenge samples. The rest of mice were challenged by intraperitoneal injection with 3×10^7 focus forming unit (FFU) of SFTSV and were observed for 28 days for signs of disease. Every 6 or 7 mice were sacrificed for their blood, livers, spleens and kidneys to be collected on day 3, 14, 21 and 28 post infections (DPI). The number of deaths from SFTSV infection was recorded daily. SFTSV loads, hematological and biochemical parameters, and histopathological evaluations were examined. Viral RNA from serum and organs (liver, spleen and kidney) was extracted using the TIANamp Virus DNA/RNA Kit and RNAprep Pure Tissue Kit (TIANGEN BIOTECH Co., Ltd., China) according to the manufacturer's protocols, respectively, and viral loads were determined by TaqMan quantitative real-time RT-PCR method using One-Step PrimeScript™ RT-PCR Kit (TaKaRa Biotechnology (Dalian) Co., Ltd., China) according to the method described previously [4]. Pathological lesions of liver, spleen and kidney were examined by hematoxylin-eosin (H&E) staining of 5-µm thick paraffin-embedded tissue sections.

PDGF-B messenger RNA expression in SFTS patients

We used ABI 7500 Real-Time PCR System (Applied Biosystems, USA). for genotyping and allele-specific gene expression assay (Realtime PCR Master Mix; Takara). We followed the manufacturer's protocol for the preparation of the PCR reactions. The primers and TaqMan probes used for the *PDGF-BB* rs1800818 polymorphism were as follows: 5′-GCTGCAAAGAGAAAACCGGAG-3′ (forward primer), 5′-GCCGACAGGTGGACGC-3′ (reverse primer), 5′-FAM-AGAGCGGCGAGCGGG-MGB -3′ (G allele-specific probe), 5′-HEX-AGAGCGGCGAGCAGG-MGB-3′ (A allele-specific probe), and the underlined base indicates the polymorphic site (referred to as GenBank accession No. NM_005427.1). Total RNA and genomic DNA were extracted from peripheral blood mononuclear cells (PBMCs) of 8 patients (heterozygous for PDGF-BB rs1800818 polymorphism) by use of Trizol (Invitrogen, USA). Complementary

DNA was prepared with oligo dT primer (Promega, USA). TaqMan assays were then performed. Finally, we measured allele-specific expression of PDGF-BB from the 8 APBMCs identified as heterozygous samples using real-time quantitative PCR. Each sample was performed in triplicate in 3 independent experiments.

REFERENCES

1. Liu W, Lu QB, Cui N, Li H, Wang LY, Liu K, Yang ZD, Wang BJ, Wang HY, Zhang YY, Zhuang L, Hu CY, Yuan C, Fan XJ, Wang Z, Zhang L, et al. Case-fatality ratio and effectiveness of ribavirin therapy among hospitalized patients in china who had severe fever with thrombocytopenia syndrome. Clinical infectious diseases : an official publication of the Infectious Diseases Society of America. 2013; 57:1292-1299.
2. Buetow KH, Edmonson M, MacDonald R, Clifford R, Yip P, Kelley J, Little DP, Strausberg R, Koester H, Cantor CR and Braun A. High-throughput development and characterization of a genomewide collection of gene-based single nucleotide polymorphism markers by chip-based matrix-assisted laser desorption/ionization time-of-flight mass spectrometry. Proc Natl Acad Sci U S A. 2001; 98:581-584.
3. Zhang X, Li X, Wu Z, Lin F and Zhou H. The p73 G4C14-to-A4T14 polymorphism is associated with risk of lung cancer in the Han nationality of North China. Mol Carcinog. 2012.
4. Jin C, Liang M, Ning J, Gu W, Jiang H, Wu W, Zhang F, Li C, Zhang Q, Zhu H, Chen T, Han Y, Zhang W, Zhang S, Wang Q, Sun L, et al. Pathogenesis of emerging severe fever with thrombocytopenia syndrome virus in C57/BL6 mouse model. Proceedings of the National Academy of Sciences of the United States of America. 2012; 109:10053-10058.

SUPPLEMENTARY FIGURE AND TABLES

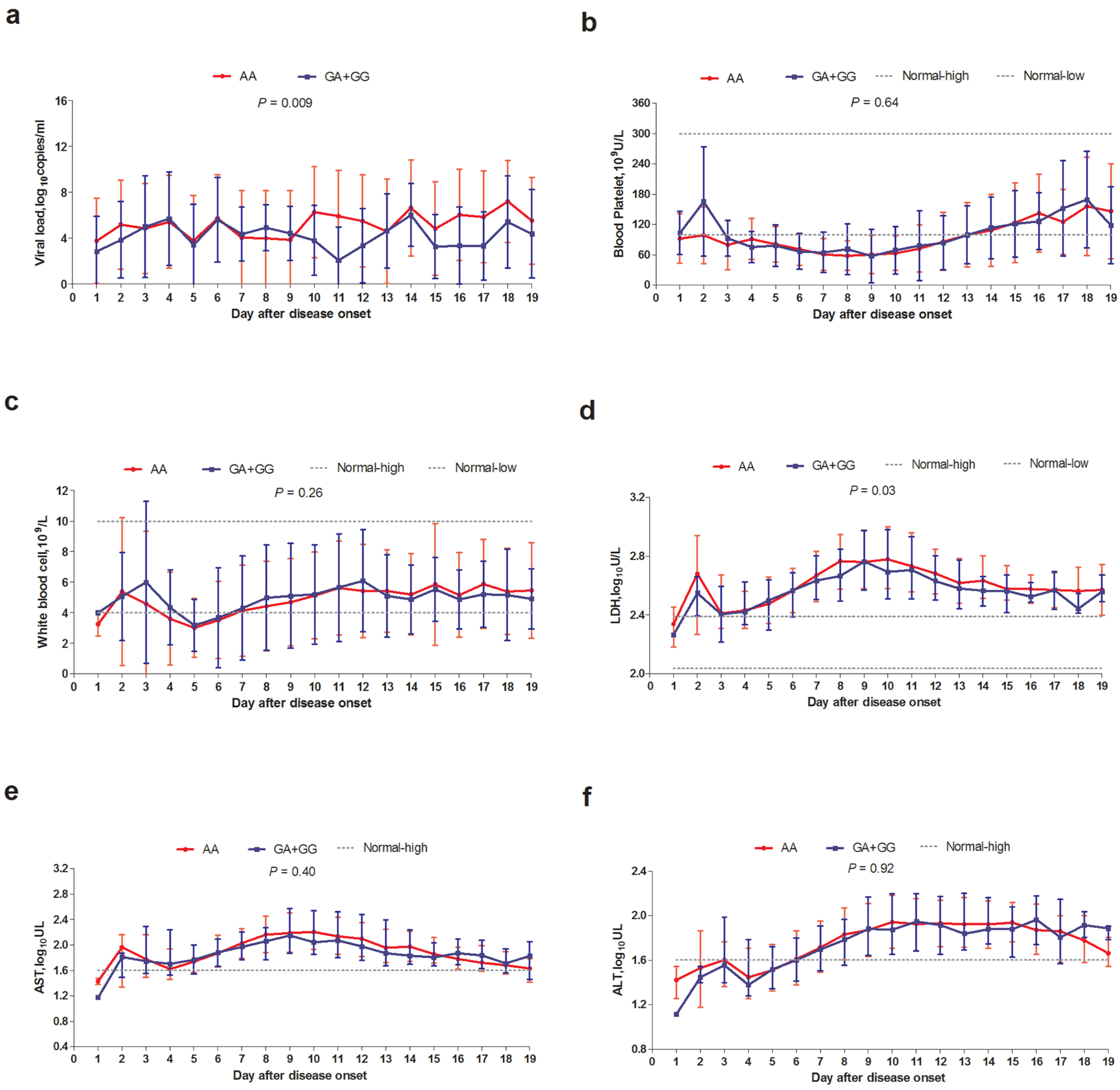

Supplementary Figure S1: Effect of *PDGF-B* rs1800818 polymorphism on dynamic profile of laboratory parameters in patients with severe fever with thrombocytopenia syndrome. a. Dynamic profile of viral load. **b.** Dynamic profile of platelets (PLT) in patients. **c.** Dynamic profile of white blood cell (WBC) in patients. **d.** Dynamic profile of lactate dehydrogenase (LDH) in patients. **e.** Dynamic profile of aspartate aminotransferase (AST) in patients. **f.** Dynamic profile of alanine transaminase (ALT) in patients. Dynamic profiles were delineated using mean and 95% confidence intervals (CIs) of each parameter. Red lines represent patients with rs1800818 AA genotype, and blue lines represent patients with rs1800818 AG+GG genotypes. The dashed lines indicate the normal level of each parameter. *P* values were computed by comparing each parameter between the two groups with the generalized estimating equation (GEE) models.

Supplementary Table S1: Primers used in sequenom platform.

SNPs	Primers	Sequences
	Extension	5'-CACATTTCAGAACCTATCTTCTT-3'
rs1800818	1st-PCR	5'-ACGTTGGATGCCGCAGAGGACGCCCAGAG-3'
	2nd-PCR	5'-ACGTTGGATGACAGGTGGACGCGGCGCGA-3'
	Extension	5'-CCCGGTCCGTCTGCCCGCC-3'
rs1800817	1st-PCR	5'-ACGTTGGATGTCCAAAGTTCACTGCAGGG-3'
	2nd-PCR	5'-ACGTTGGATGGGGCTGGTTCTTCATTCATT-3'
	Extension	5'-GATTACCTTCGCCCCC-3'

Supplementary Table S2: The genotype frequencies of *PDGF-B* rs1800817 and rs1800818 polymorphisms in patients with severe fever with thrombocytopenia syndrome and controls

SNPs and genotypes	Cases (n = 250)	Controls (n = 250)	OR (95% CI) [a]	*P* value [a]
rs1800817				
CC	215 (86.3)	214 (86.3)	Reference	
CA	33 (13.2)	33 (13.3)	1.04 (0.60-1.80)	0.95
AA	1 (0.4)	1 (0.4)	1.53 (0.09-27.09)	
CA + AA	34 (13.7)	34 (13.7)	1.05 (0.62-1.80)	0.85
rs1800818				
GG	198 (81.8)	200 (88.9)	Reference	
GT	41 (16.9)	24 (10.7)	1.79 (1.02-3.16)	0.06
TT	3 (1.2)	1 (0.4)	4.31 (0.42-43.73)	
GT + TT	44 (18.2)	25 (11.1)	1.89 (1.08-3.28)	0.022

Abbreviations: OR, odds ratio; CI, confidence interval.
[a] The ORs and *P* values were adjusted for age, sex, and underlying medical conditions.

Supplementary Table S3: Allele and genotype frequencies of *PDGF-B* rs1800818 polymorphism in mild and severe patients with severe fever with thrombocytopenia syndrome

Genotypes	Mild patients (n= 730)	Severe patients (n= 290)	Model	OR (95% CI) [a]	P value [a]
AA	572 (82.8)	226 (84)	Codominant	Reference	
AG	111 (16.1)	40 (14.9)		0.86 (0.57-1.29)	0.77
GG	8 (1.2)	3 (1.1)		0.98 (0.25-3.85)	
AG + GG	119 (17.2)	43 (16)	Dominant	0.87 (0.59-1.29)	0.48
			Recessive	1.01 (0.26-3.94)	0.99
			Overdominant	0.86 (0.58-1.29)	0.47
			Log-additive	0.89 (0.62-1.27)	0.52

Abbreviations: OR, odds ratio; CI, confidence interval.
[a] The ORs and *P* values were adjusted for age, sex, and underlying medical conditions.

Supplementary Table S4: Allele and genotype frequencies of *PDGF-B* rs1800818 polymorphism in fatal and nofatal patients with severe fever with thrombocytopenia syndrome

Genotypes	Nonfatal patients (n= 912)	Fatal patients (n= 108)	Model	OR (95% CI) [a]	*P* value [a]
AA	712 (83.1)	86 (83.5)		Reference	
AG	136 (15.9)	15 (14.6)	Codominant	0.83 (0.45-1.52)	0.44
GG	9 (1.1)	2 (1.9)		2.67 (0.54-13.27)	
AG + GG	145 (16.9)	17 (16.5)	Dominant	0.91 (0.51-1.62)	0.75
			Recessive	2.75 (0.55-13.62)	0.26
			Overdominant	0.82 (0.45-1.49)	0.51
			Log-additive	1.00 (0.60-1.68)	1

Abbreviations: OR, odds ratio; CI, confidence interval.
[a] The ORs and *P* values were adjusted for age, sex, and underlying medical conditions.
